# Analysis of the Scale of Global Human Needs and Opportunities for Sustainable Catalytic Technologies

**DOI:** 10.1007/s11244-023-01799-3

**Published:** 2023-03-11

**Authors:** Teona Taseska, Wanqing Yu, Madeleine K. Wilsey, Connor P. Cox, Ziyi Meng, Soraya S. Ngarnim, Astrid M. Müller

**Affiliations:** 1 Department of Chemical Engineering, University of Rochester, 14627 Rochester, NY USA; 2 Materials Science Program, University of Rochester, 14627 Rochester, NY USA; 3 Department of Chemistry, University of Rochester, 14627 Rochester, NY USA

**Keywords:** Climate change, Clean energy, Water–Energy–Food nexus, Sustainability, Catalysis

## Abstract

We analyzed the enormous scale of global human needs, their carbon footprint, and how they are connected to energy availability. We established that most challenges related to resource security and sustainability can be solved by providing distributed, affordable, and clean energy. Catalyzed chemical transformations powered by renewable electricity are emerging successor technologies that have the potential to replace fossil fuels without sacrificing the wellbeing of humans. We highlighted the technical, economic, and societal advantages and drawbacks of short- to medium-term decarbonization solutions to gauge their practicability, economic feasibility, and likelihood for widespread acceptance on a global scale. We detailed catalysis solutions that enhance sustainability, along with strategies for catalyst and process development, frontiers, challenges, and limitations, and emphasized the need for planetary stewardship. Electrocatalytic processes enable the production of solar fuels and commodity chemicals that address universal issues of the water, energy and food security nexus, clothing, the building sector, heating and cooling, transportation, information and communication technology, chemicals, consumer goods and services, and healthcare, toward providing global resource security and sustainability and enhancing environmental and social justice.

**Supplementary Information:**

The online version contains supplementary material available at 10.1007/s11244-023-01799-3.

## Introduction

The massive scale of global energy and manufacturing is the foremost challenge in the transition from fossil fuels to a net-zero or net-negative carbon economy. In 2019, the total global gross domestic product (GDP) was 87.57 trillion U.S. dollars [1], and the total world primary energy consumption was 581.5 × 10^18^ J (equal to 581.5 exajoules) [2]. The global energy demand was met by an energy mix that contained 80% fossil fuels [3]. Because of this high share of fossil sources, global CO_2_ emissions related to energy and industrial processes were 36.1 billion metric tons (gigatons) [4], which is causing anthropogenic climate change [5]. We analyzed data of the year 2019 because 2020 was impacted by the COVID-19 pandemic; data for 2021 were not yet available for the aspects of this analysis.

Catalysis contributes to greater than 35% of the global GDP, and catalytic processes are involved in 80% of all manufactured goods [6]. Current industrial processes are optimized in terms of cost savings, which are closely related to energy savings. Nevertheless, the most energy-intensive processes of the chemical industry possess large carbon footprints, partly owed to the enormous scale of production. In 2019, the world produced 4,100 million metric tons (megatons) of cement [7], 235 megatons of ammonia [8], 69 megatons of hydrogen via steam methane reforming plus an additional 48 megatons of hydrogen as a byproduct from other chemical processes [9], and 58 megatons of chlorine [10].

**Fig. 1:**
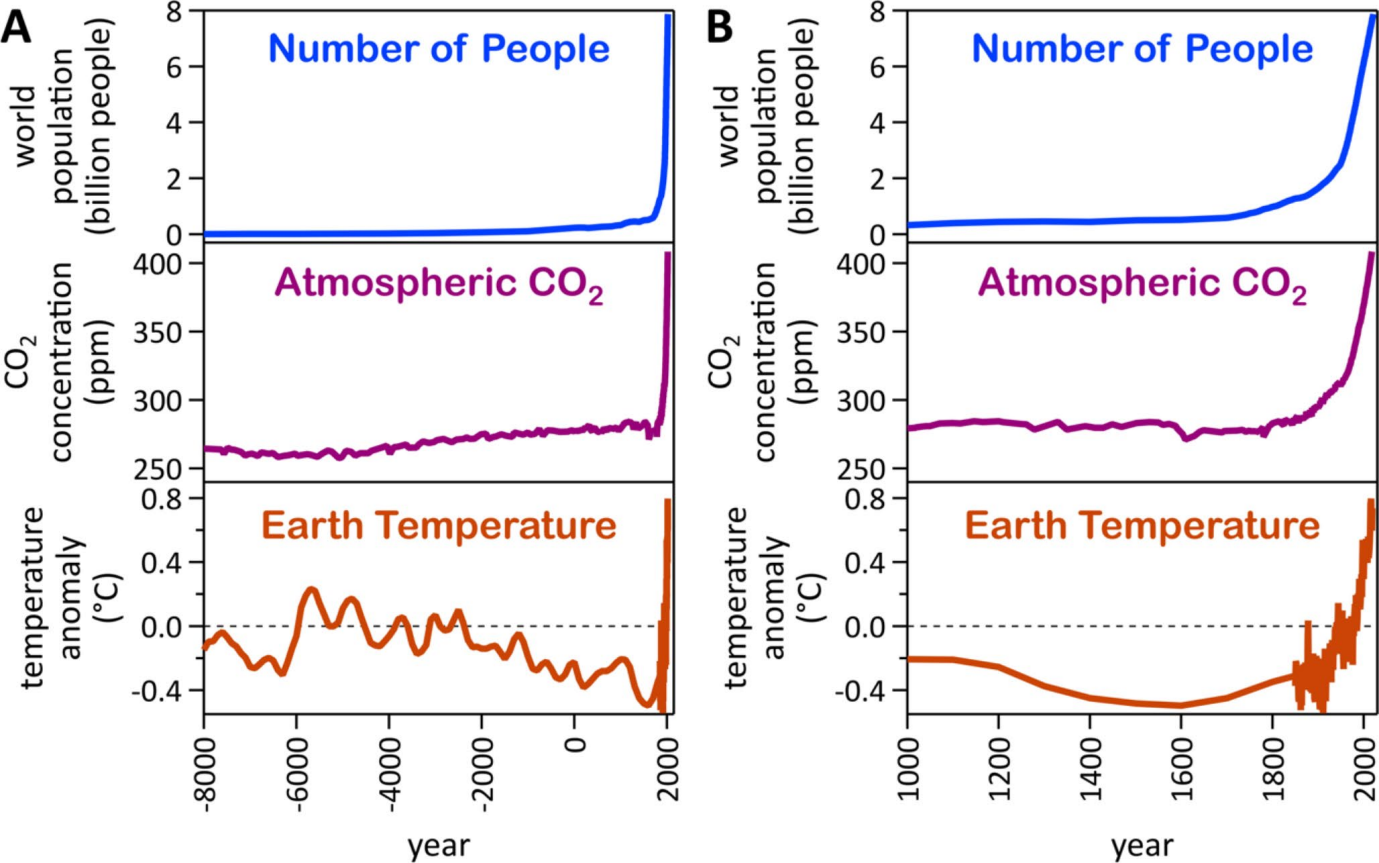
Temporal developments of the world population, global atmospheric carbon dioxide concentration, and globally averaged median temperature on the earth surface since the year 8,000 BC (A) and for the last millennium (B) [14–17]

The scale of energy, chemicals, and goods is this large because 7.7 billion people lived on the planet in 2019; currently the world population is 8.0 billion people [11, 12]. For millennia until the industrialization, the world population hovered below 1 billion people. The inventions of the steam engine, the tractor, and the Haber-Bosch process to catalytically produce ammonia as a precursor for artificial fertilizer led to explosive population growth starting in the 19th century. Since that time, atmospheric CO_2_ content grew drastically, and the earth’s median surface temperature rose concomitantly. The earth surface warms because atmospheric greenhouse gases possess greater transparency to visible radiation from the sun than to infrared radiation emitted from the planet’s surface, effectively trapping heat. The main greenhouse gases are water vapor, carbon dioxide, methane, nitrous oxide, ozone, hydrogen, and chlorofluorocarbons. Hydrogen is not infrared-active itself but acts as an indirect greenhouse gas because hydrogen gas reacts with tropospheric hydroxyl radicals to form water molecules, thus perturbing the radical-mediated decomposition of methane and ozone [13]. Comparison of temporal evolution data of the world population, global atmospheric carbon dioxide concentration, and globally averaged temperature on the earth surface clearly shows that there are *three* hockey stick curves that follow each other closely (Fig. 1).

Technological progress since the industrialization enabled unprecedented prosperity and sustained a growing world population with increased life expectancy and living standard, but simultaneously led to global warming and climate change because of anthropogenic emissions of greenhouse gases from the use of fossil fuels. Diminishing biodiversity is an additional casualty [18–20]. Hotter temperatures cause more frequent, severe, and longer heat waves, droughts, storms, and floods with catastrophic effects on humans. Sea levels rise, arctic ice caps, glaciers, and permafrost melt, precipitation patterns change towards heavy rain and snowfall events or lack of regular precipitation, and crop growing seasons shift [21]. Warmer surface temperatures create stronger tornados, cyclones, hurricanes, and typhoons more often. Destructive sand and dust storms occur with increased frequency in drought regions. More intense and frequent forest fires, extreme weather, and invasive pests and diseases threaten human livelihoods. Species are lost at an unprecedented rate of 1,000 times greater than at any other time in recorded human history [22].

The world population is expected to level off at ca. 10.9 billion people in 2100 because of progress in the education of women worldwide and related family planning decisions [11, 23]. At the current per capita carbon footprint, the maximum population size for a climate-balanced world would be ca. 3 billion people [24]; however, a sudden reduction of the world population by 5 billion people is unrealistic and would be unethical. With today’s technology, even the current and especially a growing world population causes increasing emissions, pollution, and resource depletion in a vicious cycle as long as the world economy is largely powered by fossil sources.

Therefore, humanity must transition to ethical, sustainable, and climate-friendly successor technologies that importantly are competitive with existing technologies. The Intergovernmental Panel on Climate Change (IPCC) estimates that humanity needs to not only stop adding greenhouse gases to the atmosphere but also to remove CO_2_ from the oceans and atmosphere at a rate of 10 gigatons per year by the year 2050, to limit climate change to around 1.5 degrees Celsius, a goal set by the Paris Accords [5]. Sustainable catalytic processes are emerging and will be indispensable for achieving the world’s climate goals without sacrificing the wellbeing of humans.

Here, we analyzed how global human needs are connected to energy availability and established that most challenges related to resource security and sustainability can be solved by providing distributed, affordable, and clean energy. We outline global human needs by sector and show how these needs are currently met and related to energy demand and CO_2_ emissions. We describe in detail current and future technologies, with emphasis on environmental and social justice. We highlight emerging successor technologies and catalysis approaches for solving humanity’s challenges with respect to energy, resource security, and sustainability.

## Current Technologies for Global Human Needs

**Fig. 2:**
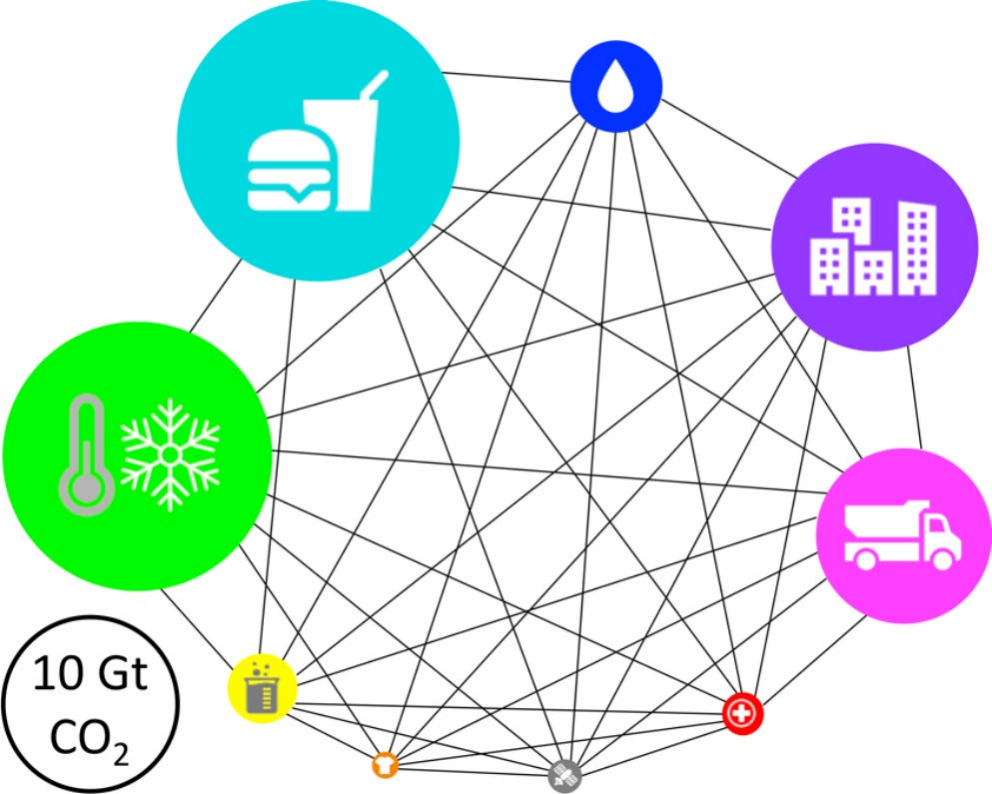
Interconnectivity of global human needs by sector. The areas of the circles represent CO_2_ emissions of individual sectors in 2019, based on available data [26–33]; the area representing an emission of 10 gigatons (Gt) of CO_2_ is depicted as a black hollow circle. Clockwise from the top, the global human needs sectors are water (blue), the building sector (purple), transportation (magenta), healthcare (red), information and communication technology (gray), clothing (orange), chemicals, consumer goods and services (yellow), heating and cooling (green), and food (cyan); Black lines indicate interconnectivities

We identified energy, water, food, clothing, the building sector, heating and cooling, transportation, information and communication technology, chemicals, consumer goods and services, and healthcare as global human needs, most of which are interconnected, and all are related to energy use and concomitant CO_2_ emissions (Fig. 2). The water–energy–food nexus is widely recognized for its interconnectivity toward achieving resource security [25]. We show in our analysis that additional sectors contribute to global human needs; meeting their requirements will provide more comprehensive resource security. We outline the enormous scale of global demands and describe the current technologies and their contribution to CO_2_ emissions per sector.

**Energy.** In 2019, the total global primary energy consumption was 581.5 exajoules [2], and the world total final energy consumption was 418.0 exajoules [34]. Primary energy consumption is the total energy demand and final energy consumption is what end users actually consume. The difference originates from the amount of energy that the energy sector requires itself and from transformation and distribution losses. Energy was provided by sources that consisted of 80% fossil fuels (26% coal, 23% gas, and 31% oil), 5% nuclear, 4% traditional use of biomass, and 10% other renewable sources [3]. Global energy-related CO_2_ emissions were 33.2 gigatons in 2019 [35]. Global CO_2_ emissions from electricity generation were 4.0 gigatons and lower than in previous years because of a higher share of renewable sources (mainly wind and photovoltaics), replacement of coal by natural gas, and higher nuclear power output; 63% of power production came from fossil sources [35]. Electricity had a share of 20% in the world total final energy consumption in 2019 [36].

**Water.** Only 2.5% of the global water resources are freshwater [37]. The total global withdrawal from natural freshwater sources was 4.0 × 10^9^ m^3^ in 2018, the most recent year of available data [38]. The global population has increased by 300% from the beginning of 20th century until now [11], and concomitantly the global water consumption has increased by 600% [39]. Access to freshwater is unevenly distributed across regions. In the U.S., 97% of the population have access to clean water, globally that number is only 74%, and in the sub-Saharan region, only 30% of the population have access to clean water [40]. Sub-Saharan Africa would require 35 billion U.S. dollars per year in capital costs, i.e. 0.5% of the total spending for global infrastructure to secure safe drinking water, sanitation, and hygiene [40]. Droughts endanger livestock and crops and threaten the wellbeing of humans through famine, increased risk of disease, and death [41]. Droughts are estimated to put as many as 700 million people at risk of being displaced as a result of water scarcity by 2030, causing mass migration [41]. Lack of water impacts more than 2.3 billion people worldwide [42].

Rising global temperatures have a detrimental impact on oceans and access to freshwater. Changing climates have always affected the water cycle because at higher temperatures more water evaporates into the atmosphere. Given the recent drastic increase of global temperatures (Fig. 1), more extreme weather events, including frequent and intense rainfall, will occur in the future, causing devastating flooding, erosion, pollution of water supplies, and algae blooms, overall limiting water access for humans and ecosystems [43]. Since the industrialization, the average global sea surface temperature has risen by approximately two degrees Celsius [44] and oceans have experienced an unprecedented acidification by increased dissolution of rising levels of CO_2_, lowering the average surface seawater pH from 8.2 before the industrial revolution to below 8.1 now, with harmful effects on ocean life [45].

Most domestic and commercial water comes from natural sources, i.e. groundwater or surface water [46]. Because of the abundance of seawater, desalination is an attractive option to provide additional clean water. Seawater desalination operates by thermal distillation (multi-effect distillation) or membrane separation (reverse osmosis). Globally, desalination processes provide a maximum capacity of 92.5 × 10^6^ m^3^ of clean water per day [47]. Desalination plants require a major seawater source, limiting the locations of facilities to coastal regions. Further, seawater desalination is energy-intensive (14–21 kWh m^–3^ for thermal distillation; 4.0–6.0 kWh m^–3^ for reverse osmosis), creating a competing water–energy nexus [48, 49].

About 71% of the Earth’s surface is covered by water, providing plenty of water as a feedstock for domestic and commercial use. Humanity has the technology to purify water and transport it to the location of usage, be it by pipelines or shipping. For example, bottled drinking water from the Fiji Islands is widely available for purchase in the U.S. [50], which is more than 12,000 km away. But water purification and transport are energy-intensive; ergo, access to clean water is, in fact, an energy problem. That means that providing distributed, affordable, and clean energy will solve the challenges related to water security and sustainability.

**Food.** In 2019, the daily per capita caloric supply was 2838 kcal globally, 3782 kcal in the U.S., 2071 kcal in sub-Saharan Africa, and 1799 in Central African Republic [51]. This means that humanity consumed globally 33 exajoules of food in one year, illustrating the enormous scale of global food supply; note that this number does not include energy spent on providing food. With current technologies, food production and its supply chain consume about 30% of the globally available energy [52]. Climate change is exacerbating the situation by putting food security at risk, especially in the developing world, because rising temperatures adversely affect water access [41] and crop yields by altering growing seasons [53]. Nowadays, 920 million people in the world lack access to a sufficient supply of nutritious and safe food [54]. Demand for food is expected to rise by 70 to 100% by 2050 because of continued population and economic growth [55].

The agriculture industry consumes globally up to 20% energy and up to 70% of the freshwater [56]. In the U.S., the farming industry uses 1.9% of the nation’s total primary energy consumption [57, 58]. Mineral fertilizers that contain nitrogen, phosphorous, and potassium are essential to enhance soil fertility and crop yields [59]. About half of the world’s population depends on the industrial production of nitrogen fertilizer from ammonia [60], which is produced by the alkali-promoted iron-catalyzed Haber–Bosch process from nitrogen and hydrogen; 235.3 megatons of ammonia were manufactured in 2019 [8]. Global nitrogen fertilizer consumption was 107.6 megatons in 2019; additionally, 46.3 megatons phosphorous pentoxide and 36.9 megatons potassium oxide fertilizers were used [59]. Apart from farming on land, fish is an important protein source. Global fish production amounted to 176.6 megatons in 2019; 52% was produced by fisheries and 48% came from aquacultures [61].

Innovations in regenerative farming, such as reducing tillage, expanding crop rotations, planting cover crops, and reintegrating livestock into crop production systems aim to address the industry’s growing carbon footprint [62]. Nevertheless, farmers and industry are reluctant to transition to more sustainable methods because of large initial investments and higher labor costs; production yields of regenerative fields are 29% lower than those of conventional farming [63].

The amount of food waste worldwide is another point of concern. In 2019, almost one gigaton of food was wasted worldwide; about 14% of food was lost between harvest and retail, and 17% of food available to consumers was discarded [64]. Wasted food accounts for 38% of total energy usage by the global food system [64].

Modern food production enabled the world’s population to grow (Fig. 1), relying on agricultural technology, innovations, and artificial fertilizers [65]. Root causes of food insecurity are conflict, extreme weather, and economic issues [66]. Food production and distribution processes are energy-intensive; therefore, food security is also an energy problem.

**Clothing.** The textile industry accounts for 5% of total global emissions because of the large world population, increased prosperity worldwide, and fast fashion [29, 67]. The fashion industry produced more than 92 megatons of waste and consumed 79 × 10^6^ m^3^ of water in 2019 [67]. Most clothing is produced from fossil-fuel-based synthetic textiles or water-intensive cotton [29]. While clothing is an essential need, the pace at which garments are acquired to keep up with the latest fashion trends is unprecedented; ca. 20 pieces of clothing per person are manufactured each year [68]. Manufacturing of clothing is energy-intensive; therefore, meeting global needs for clothing is also an energy problem.

**Building Sector.** Demand for buildings to house the world’s population is massive and continues to rise, mainly driven by population growth, economic growth, and urbanization, as well as increasing demand for cool spaces in hot regions, exacerbated by climate change. By 2050, nearly 70% of the world population will live in cities [69]. Buildings and their construction are responsible for almost one third of the total global final energy consumption and nearly 15% of direct CO_2_ emissions [70]. Globally, 4.1 gigatons of cement were produced in 2019 [71]; the largest producer was China with a 56.2% market share [72]. In addition to cement, the building sector critically depends on sand, gravel, crushed stone, and aggregates resources, which are the second most exploited natural resource in the world after water [73]. The use of sand resources has tripled in the last 20 years, reaching an estimated 40–50 gigatons per year [73]. The production of building materials and the construction of buildings are energy-intensive; therefore, meeting demands in the building sector is also an energy problem.

**Heating and Cooling.** Heating for homes, industry, and other applications accounts for about half of the total global energy consumption, significantly more than electricity (20%) and transport (30%) [74]. About 50% of total heat produced was used for industrial processes, another 46% was consumed in buildings for space and water heating and, to a lesser extent, for cooking and heating of agricultural greenhouses [74]. Cooling, i.e. air conditioning and refrigeration, requires rising energy costs, especially in light of global warming. Space cooling in buildings accounted for about 1900 TWh of electricity consumption in 2020; it is estimated that there are two billion air conditioning units used around the globe [75]. Heating and cooling consume large amounts of energy on a global scale; therefore, supplying enough heating and cooling is an energy problem.

**Transportation.** The energy demand of transportation is enormous. Transportation accounted for 36% of the final energy consumption in 2019 [34]. Oil, natural gas, and electricity were sources of energy for transportation. Of the 169 exajoules of oil that were consumed globally in 2019, 29.1 exajoules were used for road transport, 5.1 exajoules for aviation, and 1.6 exajoules for rail transport [34]. Transportation accounted for 5 exajoules of the 68 exajoules of natural gas that were consumed globally in 2019 [34]. Electricity was only a minor source of energy for transportation, consuming 1.5 exajoules of the 82 exajoules of electricity that were produced worldwide in 2019 [34]. A staggering 88% of transportation occurred on roads (58% in passenger cars, 27% as freight, 2% in buses, and 0.5% on motorcycles), whereas flights accounted for 8%, and transportation on rail and on water for 2% each [76]. Locomotion requires energy; ergo, transportation is an energy problem.

**Information and Communication Technology.** In January 2019, almost 4.4 billion people accessed the internet [77], and 5.5 billion smartphones were in use across the world [78]. The International Energy Agency estimates that the carbon footprint for one hour of streaming video in 2019 was 36 g CO_2_ [79]. In 2017, more than one billion hours of YouTube content was watched every day [80], resulting in the emission of more than 1.3 megatons CO_2_ per year just from YouTube streaming. In addition to the downloads, ca. 30,000 h of new content was uploaded on YouTube per hour in 2019 [81]. Digital content is indispensable to raise awareness about current affairs, including to convince the public that anthropogenic global warming is indeed happening (Fig. 1). Nevertheless, consumption of digital content contributes to CO_2_ emissions, which in turn exacerbate the world’s climate change issues. Other digital services, such as streaming digital content from other providers, social media, videoconferencing, messaging, cell service, search engines, ecommerce, data storage, artificial intelligence, academic research, digital healthcare, government and defense applications, and bitcoin mining, add to the enormous energy demand and concomitant carbon footprint of information and communication technology. It is estimated that Google, Facebook, Microsoft, and Amazon together store at least 1,200 petabytes of information; this figure does not include other storage and cloud providers or the massive servers of governments, academia, and industry [82]. The entire digital universe was estimated to contain 44 zettabytes of data in the beginning of 2020, and the amount of data in the internet created each day is estimated to reach 463 exabytes by 2025 [83]. We note the lack of public availability of total energy consumption and climate impact data for the information and communication technology sector. The generation, storage and delivery of digital content requires energy; therefore, meeting demands in the information and communication technology sector is also an energy problem.

**Chemicals.** The manufacturing of chemicals happens on a large scale. In 2019, the total global revenue of the chemical industry was 4.0 trillion U.S. dollars [84]. Based on revenue, the largest chemical company is BASF in Germany [85], with a revenue of 63 billion U.S. dollars in 2019 [86]. Sales in U.S. dollars consisted of ca. 14 billion in surface technologies, 12 billion in materials, 10 billion in chemicals, 9 billion in industrial solutions, 8 billion in agricultural solutions, and 6 billion in nutrition and care products [87]. Global direct CO_2_ emissions from primary chemical production were 941 megatons of CO_2_ in 2019 [88]. The manufacturing of chemicals is energy-intensive; as a result, supplying chemicals for the world is largely an energy problem.

**Consumer Goods and Services.** The scale of global consumer goods and services is enormous. Consumer goods are products sold to individuals to satisfy their own needs and wants, without enabling further economic production activity. Consumer goods and services are a major component of the GDP and global trade. Consumer goods are categorized as durable (typically lasting at least 3 years) and nondurable (shelf live of less than 1 year). Durable goods, also called slow moving consumer goods, such as automobiles, appliances, furniture, tableware, tools and equipment, sports equipment, luggage, electronics and software, musical instruments, books, and jewelry, are not required on a regular basis or not purchased new. The global durable goods wholesalers market size was 20.5 trillion U.S. dollars in 2021; wholesalers provide products to retailers [89]. In the U.S., durable goods accounted for 9% of the GDP in 2019, totaling 1.8 trillion U.S. dollars [90]. Nondurable goods, also called fast moving consumer goods or consumer packaged goods, such as groceries, medicines, cleaning supplies, personal care products, and office supplies, had a market size valued at 10.0 trillion U.S. dollars in 2017 and are projected to reach 15.4 trillion U.S. dollars by 2025 [91]. Services accounted in 2019 for a share of 65% of the global GDP, resulting in a total global market value of 56.7 trillion U.S. dollars [1, 92]. Carbon dioxide emissions of consumer goods and services are reported together with those of transportation due to trade and emissions from the energy and food sectors [93]. The globally averaged per-capita consumption-based CO_2_ emissions were 4.76 tons in 2019 [94], resulting in 36.7 gigatons of CO_2_ emissions. The global supply of consumer goods and services critically depends on the availability of energy, which renders the consumer goods and services sector also an energy problem.

**Healthcare.** Demand for healthcare is rising because of population growth, economic growth, and efforts towards health equity, especially in Africa, where less than 50% of the people have access to modern health facilities [95]. Healthcare cost cannot be used to quantify global healthcare availability and quality because cost is not related to health outcomes. Health expenditures in the U.S. are far greater than in other high-income countries, but Americans experience worse health outcomes than their international peers [96]. Healthcare globally accounts for 4% of CO_2_ emissions, more than the aviation or shipping industries; the healthcare supply chain, i.e. production, transport, use, and disposal of goods and services, accounts for the greatest share of emissions (71%) within the healthcare sector [95]. Healthcare critically depends on medical advances and an educated workforce; some aspects of healthcare can only be met by a stable energy supply.

## Sustainable Successor Technologies

After we analyzed the enormous scale of global human needs and outlined existing technologies, including their energy demand and climate impact per sector (Fig. 2), we highlight opportunities for catalytic successor technologies to provide global resource security, sustainability, and clean energy.

Sustainability has different meanings in different circumstances. In the context of future global resource security, sustainability has been defined as the “long-term preservation of opportunities to live well” [97]. Curren and Metzger stated that “the language of sustainability is a way of referring to the long-term dependence of human and nonhuman well-being on the natural world in the face of evidence that human activities are damaging the capacity and diminishing the accumulated beneficial products of the natural systems on which we and other species fundamentally rely” [97]. This means that any solution to limit climate change must proceed without interfering with the wellbeing of humans and other life forms; drastic reduction of the number of people on Earth or Stone Age living standards are not an option. Therefore, humanity must transition away from the large-scale use of fossil fuels toward ethical, sustainable, and climate-friendly successor technologies that are affordable, safe, convenient, globally scalable, and generally competitive with existing technologies.

The most abundant, distributed, globally scalable, affordable, and cleanest source of energy is the sun. Solar energy is carbon-neutral after amortization of the capital and operational carbon footprints of facilities that produce electricity, such as wind turbines and photovoltaic solar farms. Current efforts aim at increasing the share of solar sources to enable a growing supply of affordable, renewable electricity [35]. However, the local intermittency of solar radiation requires storage of solar energy. Nuclear energy generates electricity without the use of fossil fuels, but there are fundamental engineering and resource scaling limits [98]. Therefore, the development of new successor technologies for the conversion and storage of solar energy is urgently needed.

Existing solar energy capture technologies, mainly wind and photovoltaics, generate power increasingly efficiently and economically [99]. In fact, the world’s best solar power schemes now offer the most affordable electricity in history, i.e. utility-scale solar power is cheaper than coal and gas powered electricity production in most major countries [99]. Cost-effective, clean electricity provides extraordinary opportunities for electrocatalytic processes, which have several advantages over conventional manufacturing. Electrocatalysis proceeds at ambient to moderate pressures and temperatures, substantially lowering energy demand compared to thermochemical processes. Electrocatalysis does not require large facilities, thus lowering capital expense and enabling operation in mobile or distributed units. Finally, the biggest advantage of electrocatalysis is that it can sustainably be powered by renewable electricity [100, 101].

**Fig. 3:**
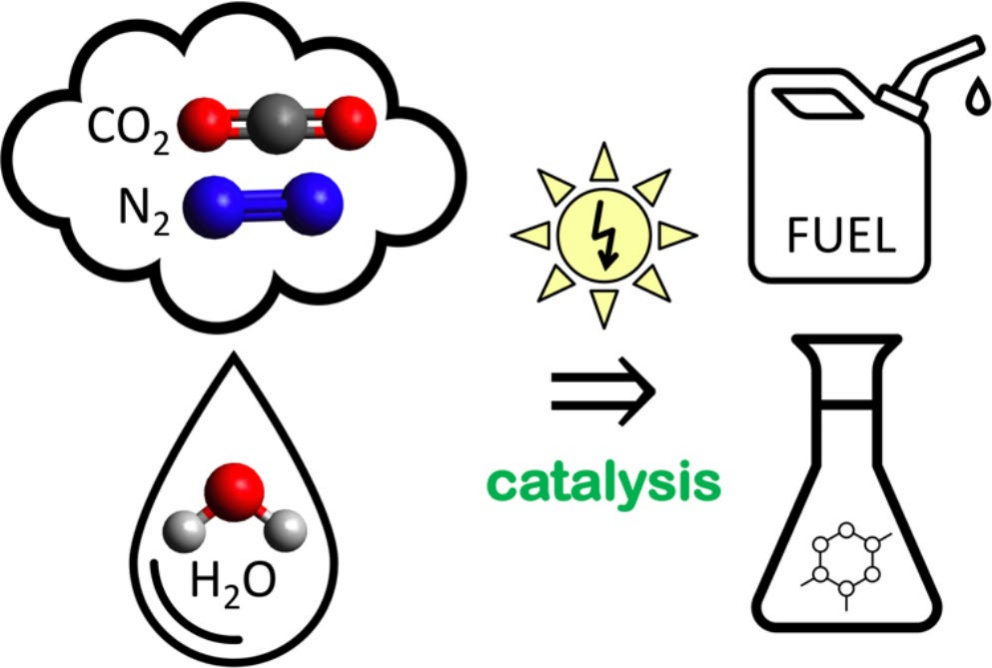
Sustainable catalytic manufacturing of fuels and commodity chemicals from abundant, small-molecule feedstocks, i.e. water, carbon dioxide, and nitrogen, powered by solar electricity

Electrifying everything replaces the fossil fuel economy with climate-friendly alternatives. Large-scale electrochemical processes, such as the Hall–Héroult process for smelting aluminum, the chloralkali process to produce chlorine and sodium hydroxide, and water electrolysis to generate hydrogen, have been an integral part of the chemical industry for decades. New efficient electrocatalytic processes must be developed for fuels and other chemicals through science-based material and process design. The electrocatalytic production of e-fuels and commodity chemicals must be accompanied by a drastic increase of the share of renewable electricity in the total energy supply to achieve carbon neutrality [102]. The world needs a paradigm shift toward more bottom-up manufacturing from our most abundant, small-molecule feedstocks, i.e. water, carbon dioxide, and nitrogen, instead of the current top-down approach of cracking oil and coal for the production of fuels and valorized chemicals (Fig. 3). Utilization of atmospheric or ocean CO_2_ as a carbon feedstock results in net-carbon-negative processes. The transition from fossil sources to sustainable electrocatalytic technologies requires the development of new processes and catalysts that must be durable, efficient, selective for a single product, nonprecious, and nontoxic for economic viability and widespread use [101, 103, 104].

**Fig. 4:**
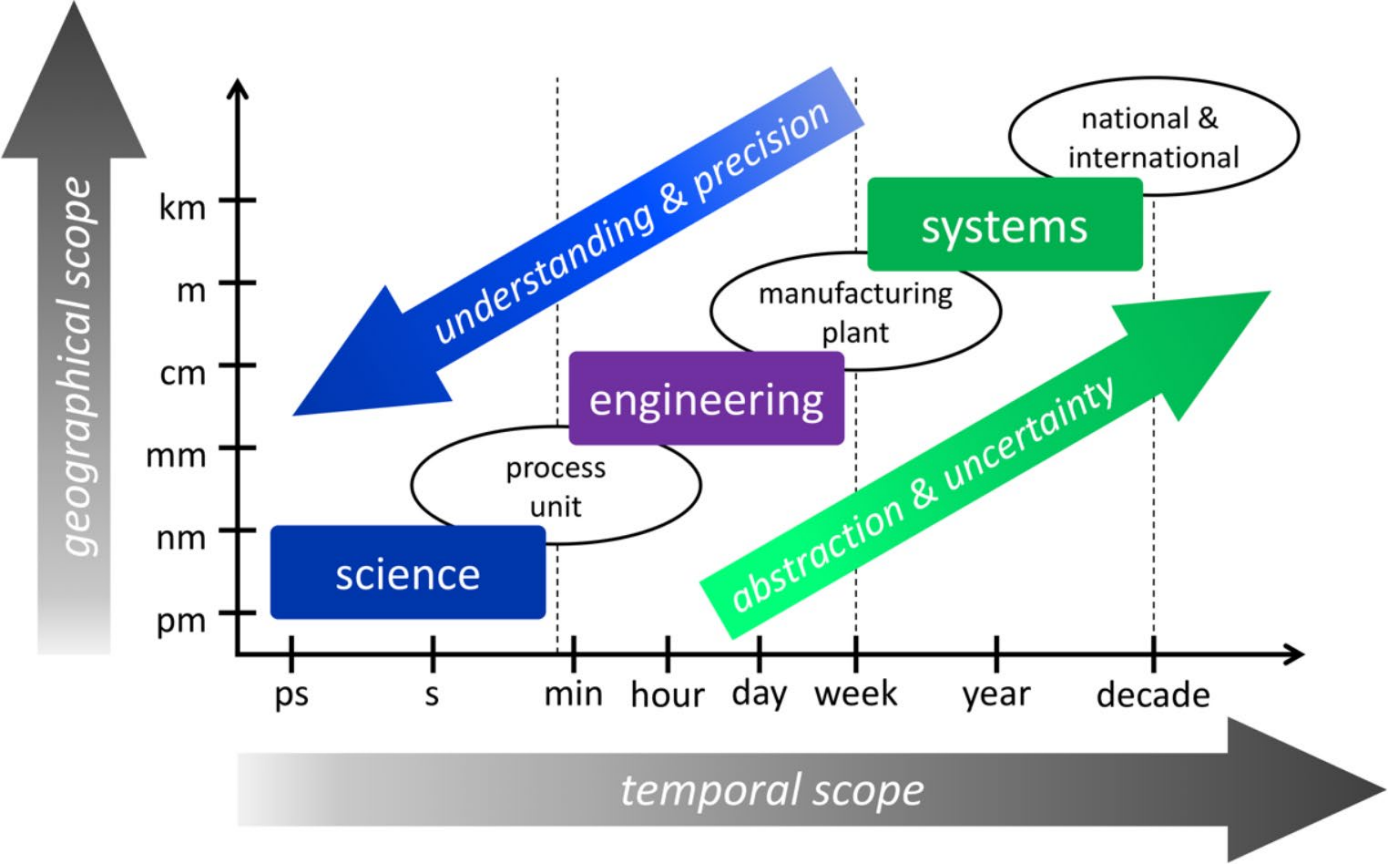
Complexity of sustainable manufacturing and energy systems from scientific and engineering solutions to national and international systems

The catalytic Haber–Bosch process together with the industrial revolution enabled unprecedented value generation, prosperity, and explosive population growth in the last century, which caused anthropogenic climate change (Fig. 1). It is the social responsibility of catalysis scientists and engineers to make a difference in the decarbonization of the world economy. Changes in value chains are required to decarbonize fuels and make manufacturing more environmentally friendly. Viable opportunities for electrocatalysis driven by renewable electricity exist already upstream in the value chain, whereas thermochemical catalysis processes are still required in downstream chemical manufacturing. Decarbonization is a complex, multiscale, and multidisciplinary challenge that requires a multi-faceted approach. Life cycle analyses of the environmental impacts of systems with varying complexity show that innovation impacts must be shifted across the entire value chain [105]. Environmental benefits at the scientific scale must translate to the global scale [106] (Fig. 4). Decarbonization of the economy is a multi-objective optimization problem that is characterized by tradeoffs. Efficient optimization requires the development of techniques to generate Pareto frontiers within each framework of objectives for a given system [107], which must guide research and development of scaled-up low-carbon processes and products.

Research and development of new materials and catalytic processes must happen now with utmost urgency because of the enormous climate impact of continued use of fossil sources. Scientists and engineers all over the world are called upon to dedicate their careers to solving humanity’s grandest challenge of providing a livable Earth for future generations. Academia and industry must partner and coordinate fundamental and applied science and engineering to accelerate the transition to sustainable energy and manufacturing. Raising public awareness about the massive scale of the challenges and the catastrophic impact if nothing is done will create the political will for change. Innovations must be coupled with policies because politics drive research and development. Clear, stable, consistent government frameworks that are homogeneous across the world are needed for the compatibility of decarbonization efforts with global trade. The current uncertainties of evolving environmental impact mitigation policies and associated costs are a major obstacle in industry projections toward sustainable manufacturing. Serious investments in research funding in academia and industry for emerging and future solutions are needed; for example, in the U.S., federal funding for the development of sustainable successor technologies is still substantially less than for medical research. Government appropriations must significantly increase to enable fundamental research that is key to accelerate the discovery of new catalysts and climate-friendly catalytic processes.

In the following, we outline ethical short- to medium-term approaches, i.e. immediately to the next 30–40 years, and long-term solutions to provide resource security and sustainability on a global scale.

**Short- to medium-term solutions.** Immediate efforts mainly center on conservation of energy and resources. Industrial processes are often optimized with regard to energy and resource intensity to lower cost, but conservation at the level of personal choice still has a large impact. Fuel switching from carbon-intensive coal to natural gas, which is the least carbon-intensive fossil energy source, for electricity generation reduces CO_2_ emissions per kWh by 45% [108]. The energy share of zero-emission power sources, such as nuclear fission, wind, and solar photovoltaics, is steadily increasing and accounts now for 27% of world energy usage [109]. Medium-term initiatives, i.e. in the next three to four decades, focus on emerging technologies that lower greenhouse gas emissions and are amenable to distributed use to minimize energy spent on transportation.

**Energy.** The total global primary energy consumption is projected to reach 880 exajoules, equivalent to 244 TWh, by 2050 [110]. Shifting energy sources to zero-carbon renewables and nuclear energy, as well as replacing coal by natural gas for electricity generation lowers CO_2_ emissions [108, 109]. Energy efficiency and conservation are important avenues to reduce energy consumption via personal choices of consumers [111]. The main efforts include buying energy-efficient products and vehicles with high fuel economy, using energy control systems in household, commercial, and industrial facilities, and changing personal habits, such as turning off lights and electric appliances when not in use [111]. Replacement of household items and appliances with more energy efficient ones, such as light-emitting diodes (LED) instead of incandescent lightbulbs, programmable instead of static thermostats, smart power strips, and energy efficient windows, saves energy. For example, electricity used by electronics that are turned off or in standby mode accounts for 75% of the energy used to power household electronics. This energy waste is preventable by using smart power strips that turn off at programmable times, through remote switches, or based on device status [112]. As a result of these measures, U.S. energy use did not increase since the year 2000, despite economic growth of ca. 30% [113]. Energy efficiency and conservation efforts have done more to meet the U.S. energy demand than oil, gas, and nuclear power over the past 40 years [113].

**Water.** Conservation of water improves water shortages. Consumers can stop leaks, buy water-saving appliances, install flow restrictors, and plant water-saving plants [114]. Developing better daily habits, such as taking shorter showers and turning off the water while brushing teeth or washing dishes, also help. Water-saving features are able to reduce domestic water use in the U.S. by 35% [115]. Conservation alone will not solve global issues with access to water. Seawater desalination is an attractive technology to provide clean water in addition to surface freshwater or groundwater, especially to offset the effects of droughts. As of now, the cumulative global installed capacity of seawater desalination processes is 92.5 × 10^6^ m^3^ of clean water per day [47]. About 3.2 kWh per cubic meter of water is needed for distribution via water lines or shipping [116]. Capacitive deionization has emerged as a technology with a lower energy demand (ca. 1 kWh m^–3^) than traditional seawater desalination, but the method is limited to brackish water with low to medium salinity, and parasitic reactions and relatively low desalination capacity impede the efficiency and reliability needed for long-term commercial use [117]. Atmospheric water generators are emerging to utilize an estimated 12.9 × 10^12^ m^3^ of renewable water in the atmosphere [118]. They have the advantage that they do not require access to surface water sources and ergo can work on dry land, even in remote locations. Atmospheric water generators have the potential to produce 20 L (household system) to over 10,000 L (commercial system) of clean water per day [119]. However, the energy-intensity of atmospheric water generators depends on the humidity and temperature of the surrounding environment. At cold and dry conditions, atmospheric water generation systems do not work, limiting universal and widespread use of this technology [120, 121]. New electrocatalytic water purification technologies are emerging. For example, electrochemical synthesis of hydrogen peroxide from water generates purified water, is amenable to decentralized use in remote locations, and can be powered by renewable electricity [122]. Likewise, electrocatalytic water splitting and use of hydrogen and oxygen in fuel cells produce clean water [123–130].

**Food.** Reducing the large carbon footprint of food [131] focuses on eliminating food waste and lowering CO_2_ emissions associated with food production. Wasted food accounts for 38% of total energy usage by the global food system [64], and one third of all food in the U.S. remains uneaten [132]. Strategies to prevent food waste are impactful and include better planning of meals and food purchases, better storage of food ingredients, repurposing prepared food, and donations of surplus food [133]. Food waste in the supply chain can be reduced by local production and consumption, which shortens transportation paths, intelligent packaging, increasing shelf life, increasing the number of hubs and decision points in the supply chain, and big data analysis of supply chain decisions [134]. Technologies to reduce CO_2_ emissions associated with food production include the use of genetically modified, more efficient crops [135] and application of proper amounts of fertilizer, which also prevents harmful eutrophication of water bodies by runoff of excess fertilizer [136]. Emerging solutions to make fertilizer production environmentally safer are the replacement of fossil-fuel-derived hydrogen with green hydrogen from electrochemical water splitting [137] or emerging electrocatalytic ammonia production [138–140].

**Clothing.** Fashion consumption creates large amounts of waste. Therefore, sustainability efforts are mostly aimed at slowing the pace of fast fashion by raising public awareness and influencing consumer habits [29, 67].

**Building Sector.** Buildings and their construction account for almost one third of total global final energy consumption and nearly 15% of direct CO_2_ emissions [70]. Zero-energy buildings combine energy efficiency (innovative insulation, window shading, and smart windows [141]) and renewable energy generation (mainly solar photovoltaics and geothermal heating and air conditioning) to mitigate the energy crisis [142]. Greener building materials, such as wood, bamboo, cork, or cob, albeit flammable and less durable, have a favorable carbon footprint compared to cement, are good thermal insulators and lightweight, which reduces transportation emissions [143].

**Heating and Cooling.** Systems for heating and cooling are well developed; modern all-electric furnaces or boilers reach efficiencies of 95 to 100% [144], but use grid electricity. Geothermal heat pumps do not rely on fossil fuels, and homeowners can save 30 to 70% on heating costs and 20 to 50% on cooling expenses, compared to conventional systems [145]. Minimizing energy losses from heating and cooling of buildings relies on innovative insulating and window materials [141]. Emerging heating technologies for industrial manufacturing processes are electric arc furnaces and heat pumps [146]. Several new technologies are emerging for alternative refrigeration: magnetic [147, 148], electrocaloric [149], thermoelectric [150], thermoacoustic [151], Stirling [147, 152], barocaloric [153], and elastocaloric [154] refrigeration methods are being developed.

**Transportation.** In 2019, 51% of global transportation occurred in passenger cars [76]. Conservation measures center therefore on incentives for carpooling, bicycling instead of driving, and public transport instead of individual vehicles, as well as public awareness campaigns toward driving less distance. Buying locally produced goods reduces transportation-related energy expenditures. However, transportation enables global trade, which is expected to continually increase [155]. Sustainable successor technologies for transportation focus on increasing the share of electric vehicles, which have zero tailpipe emissions and thus help mitigate local air pollution issues in addition to decarbonization benefits. Although the production of batteries for electric vehicles is more energy intensive than that of combustion engine vehicles, the greenhouse gas emissions associated with an electric vehicle over its lifetime are typically lower than those from an average gasoline-powered vehicle [156]. Increasing the share of nuclear energy and renewables for electricity generation together with a rising share of electric vehicles will reduce the carbon footprint of transportation. Fuel cell technologies are emerging to power clean vehicles with longer ranges than battery-powered vehicles [129, 130].

**Information and Communication Technology.** Enabled by technological innovations, societies all over the world rely increasingly on the digital world. As a result, the energy consumed by data centers accounts for about 1.3% of the world’s total electricity usage [33]. Additional energy is consumed by personal computers and devices. Total energy consumption data for the information and communication technology sector are not publicly available, impeding quantitative analyses of potential decarbonization solutions. In data centers, about 40% of the total energy is consumed for cooling the equipment, whereas only 45% is used for actual computing tasks [157]. Data center waste heat has been used to heat homes in the surrounding areas [158]. Nevertheless, new technologies are needed to reduce the energy intensity and associated carbon footprint of the information and communication technology sector. Quantum computers have emerged as a solution to drastically increase computing efficiency, requiring significantly less energy (ca. 25 kW) than modern classical supercomputers with comparable computing power (1–10 MW) [159]. Ambient condition superconductors are being researched and have the potential to eliminate most heat losses because of resistance-free electrical conduction [160, 161].

**Chemicals.** Reducing carbon emissions in the chemical industry focuses on innovations in process and chemical engineering, including utilization of big data and supercomputing, and advances in materials science, process design, sensors, analytics and catalysts [162]. Electrification of the chemical process industries is gradually increasing. Electric arc furnaces, heat pumps, and electrolysis processes contribute to sustainable manufacturing [146]. Hydrogen has an energy storage density of 39 kWh per kg [163]. Hydrogen production by water splitting currently accounts for less than 0.1% of global dedicated hydrogen production [164]. Declining costs for renewable electricity enable the scaleup of clean water electrolyzers toward a green hydrogen economy [164]. Engineering solutions must be developed to prevent hydrogen leaks because hydrogen is a potent indirect greenhouse gas [13]. New catalysts and processes are needed to replace current thermochemical catalysis processes by electrocatalysis in upstream chemical manufacturing. Importantly, electrocatalytic processes must be efficient and cost-effective, especially with respect to capital expense because losses due to obviating existing capital must be offset. Process intensification as well as catalyst recovery and circularity improve chemical manufacturing sustainability. Emerging technologies to produce commodity chemicals and fuels are electrocatalytic conversion of hydrocarbons [165, 166] and CO_2_ reduction electrocatalysis [101, 167–170]; direct capture of CO_2_ from the air or oceans renders the process net-carbon-negative [171]. Likewise, in artificial photosynthesis, electrocatalysts powered by sunlight convert captured atmospheric CO_2_ together with photoelectrochemically produced hydrogen from water splitting to valorized chemicals [172–178].

**Consumer Goods and Services.** The chemical industry is involved in 96% of all consumer goods [179]. Ergo, reductions in CO_2_ emissions by the chemical industry will simultaneously decrease the carbon footprint of consumer goods. Consumer services include the hospitality industry, leisure activities, travel, transportation, information technology, media, ecommerce, finance, insurance, leasing, utilities, events, and culture. Consumer services needs are related to the transportation, information and communication technologies, energy, and building sectors. Associated conservation measures and emerging successor technologies are described in those sectors above.

**Healthcare.** Healthcare globally accounts for 4% of CO_2_ emissions, mainly from the healthcare supply chain, i.e. production, transport, use, and disposal of goods and services [95]. Reduction of the carbon footprint in the healthcare sector center therefore on energy savings and the transition to sustainable successor technologies in the transport and goods and services sectors, as well as reducing waste by recycling.

In Table 1, we compare the technical, economic, and societal advantages and disadvantages of short- to medium-term solutions to reduce CO_2_ emissions, organized by sector, to gauge the practicability, economic feasibility, and likelihood of widespread use of successor technologies on a global scale.

**Table 1:** Advantages and disadvantages of sustainable successor technologies, organized by sector

Sector	Technology	Advantages	Ref.	Disadvantages	Ref.
Energy	Electricity generation using natural gas	• Natural gas is abundant • Lower greenhouse gas emissions compared to other fossil fuels • Cheaper than other fossil fuels	[180]	• Natural gas is a fossil fuel and contributes to climate change • Transportation of natural gas releases methane, which is a more potent greenhouse gas than carbon dioxide • Environmental issues with sourcing – fracking is the most commonly used method	[180]
	Light-emitting diodes	• More efficient to use • Have a long life • More reliable • Are available in a variety of colors	[181]	• More expensive • Not robust against high voltage spikes • Little effective wide-area • Cause backward heat dissipation	[181]
	Programmable thermostats	• Can save energy • One-time setup • Flexible operational settings	[182]	• High initial cost • Not compatible with all heating and cooling systems • Depend on user habits and do not always save energy	[182]
	Smart power strips	• Prevent devices from wasting electricity • Extend the life of devices • Protect against power surges	[183, 184]	• Can be hijacked or hacked to damage devices	[184]
	Energy efficient windows	• Save money • Reduce the carbon footprint • Have better insultation, which improves temperature control and reduces noise from forced air systems • Provide protection against the sun’s ultraviolet rays • Require less maintenance	[185]	• Higher initial cost than conventional windows	[185]
Water	Seawater desalination	• Provides drinking water in areas where natural supply of potable water is limited • Reduces pressure on freshwater supplies	[186]	• High costs to build and operate a desalination plant • Environmental issues with the disposal of brine • Uses or produces chemicals, such as chlorine, carbon dioxide, hydrochloric acid and anti-scalents, that can be harmful in high concentrations	[186]
	Capacitive deionization	• Allows salt removal at low pressures and room temperatures at small cell voltages • Direct transportation of ions out of the feed allows potentially highly energy efficient desalination of brackish water • High energy recovery is possible • Low operational cost	[187]	• Limited by the availability of sustainable electrode materials that have high electro-adsorption capacity and high average salt absorption rate • Limited to the desalination of brackish water, cannot be used for seawater	[187]
	Atmospheric water generators	• Do not require access to surface water sources, so they can work on dry land • Low capital and operational expenses • Useful for emergency and medical purposes	[119]	• Energy intensity depends on the humidity and temperature of the surrounding environment, limiting operation in cold and dry conditions • High initial cost	[120]
	Electrochemical synthesis of hydrogen peroxide from water	• Amenable to decentralized use in remote locations • Can be powered by renewable electricity • Cost-effective and applicable on both large and small scales	[122, 188]	• No field testing yet • The development of catalysts for this process is challenging because many electrode materials favor competing reactions	[188]
	Electrocatalytic water splitting	• One of the most promising clean energy solutions • Excellent catalytic activities and efficiencies have been reported • Uses water, which is an abundant and renewable source	[189]	• Large-scale production is inhibited by high electricity cost, capital investment, and electrolysis media • Usually very harsh conditions (strong acid/alkaline) are used, which cause issues with corrosion, catalyst stability, and technical difficulties with the membrane	[189]
	Hydrogen–oxygen fuel cells to produce clean water	• Does not use or produce pollutants • High efficiency • Currently used in spaceships	[190]	• Very expensive because of noble metal catalysts • Requires large compressed-gas cylinders to store and transport hydrogen and oxygen • Hydrogen is an explosion hazard	[190]
Food	Local production and consumption	• Shortens transportation paths • Lower emissions • Supports the local economy • Can avoid the exploitation of workers in developing countries	[191]	• Limited variety • Might be more expensive, which might not be affordable for many people • Might result in increased unemployment in the conventional food industry • Might decrease exports • Might cause economic problems for developing countries	[191]
	Use of genetically modified, more efficient crops	• Increased pest resistance and herbicide tolerance • Might be more nutritious • More economical	[192–197]	• Can distribute foreign genes in the environment • Can generate pesticides that will lead to tougher pests • Can cause allergic issues for humans • More expensive compared to non-genetically modified crops	[192–197]
	Application of minimum amounts of fertilizer	• Prevents harmful eutrophication of water bodies by runoff of excess fertilizer • Plants get the proper nutrients and develop ideally • Builds healthy soil	[136]		
	Electrocatalytic ammonia production	• Environmentally friendly alternative to the energy-intensive Haber-Bosch process • Promising method of storing renewable energy in chemical bonds • Allows production under milder conditions in small-scale, distributed, and on-site electrolysis cells • Can be powered by renewable electricity • Can directly utilize water as the hydrogen source	[198]	• Emerging technology, not field tested • Expensive catalysts with low activity and selectivity • Potential durability issues • Competing hydrogen evolution in aqueous systems	[198]
Clothing	Reducing the pace of fast fashion	• Lowers emissions	[29, 67]	• Threatens employment opportunities • Infringes on artistic freedoms	[29, 67]
Building sector	Zero-energy buildings	• Energy efficient via innovative insulation, window shading, and smart windows • Renewable energy generation via solar photovoltaics, geothermal heating, and air conditioning • Reduced monthly living expenses • Environmentally friendly • Reduces carbon dioxide emissions • Higher resale value	[141, 142, 199]	• Higher initial cost • Not suitable for regions with colder temperature • Limited availability of experienced designers	[199]
	Greener building materials (bamboo, cork, or cob)	• Favorable carbon footprint compared to cement • Good thermal insulators • Lightweight • Reduce transportation expenses • Reduce the long-term cost • Improvement in indoor air quality	[143, 200, 201]	• Higher initial build cost than cement • Issues with availability depending on location • Building maintenance might be difficult • Unclear long-term effects • Flammability • Less durable than cement	[200, 201]
Heating and Cooling	Geothermal heat pumps	• Do not rely on fossil fuels • 30 to 70% less operational cost (heating) than furnaces • 20 to 50% less operational cost (cooling) than traditional air conditioners • Do not release any harmful chemicals into the air • Safe to use in hot climates	[145, 202, 203]	• High capital and installation expense • Must be installed on a concrete slab • Require high amount of maintenance • Not recommended in locations where the ground freezes	[203, 204]
	Innovative insulating and window materials	• Reduce energy requirements • Decrease carbon footprint • Can reduce noise pollution • Improve the aesthetics of buildings	[205, 206]	• Can create fog on the glass • Can keep the heat inside during the summer • Very challenging to replace	[205, 206]
	Magnetic refrigeration	• Environmentally friendly	[207]	• Low efficiency	[207]
	Electrocaloric refrigeration	• Energy efficiency has the potential to exceed 65%	[208]	• Emerging, research needed to discover compact structures that can create high electric fields	[208]
	Thermoelectric refrigeration	• Lightweight • More storage capacity • Does not use refrigerants, ergo no chemical environmental issues • Can run in reverse, to act as a heat pump • Quiet because of operation without a compressor	[209]	• Limited by ambient temperature because of heat dissipation from the hot side of the Peltier element • More expensive than conventional refrigerators	[209]
	Thermoacoustic refrigeration	• Does not have any moving parts • Does not require the use of harmful refrigerants	[210]	• Uses helium • Low coefficient of performance and efficiency	[210]
	Stirling refrigeration	• Can directly run on any available heat source • Very flexible • Operates at low pressure • Works easily and more efficiently in cold weather	[211]	• Requires heat exchangers • Requires large temperature differences for high efficiency • Complicated dissipation of waste heat	[211]
	Barocaloric refrigeration	• Promising new technology for energy saving because its performance is higher than vapor compression • Works using the barocaloric effect • Uses solid-state caloric materials as refrigerants	[212]	• Emerging, no clear assessment of viability yet	[212]
	Elastocaloric refrigeration	• Higher efficiency than vapor compression refrigeration • Does not use gases or volatile liquids • Not hazardous • Contains reusable and recyclable components	[213]	• Requires very high pressures • Expensive • More research needs to be done on material, system integration, and heat transfer engineering	[213]
Transportation	Carpooling	• Reduces pollution and greenhouse gas emissions • Less expensive than single-occupancy driving • Reduces traffic • Brings people together	[214]	• Less flexible • Safety concerns for vulnerable populations • Health concerns regarding communicable diseases • Insurance issues for the driver	[214]
	Bicycling	• Reduces fossil fuel consumption • Improves cardiovascular health • Inexpensive • Easy to learn	[215]	• Bicycle injuries and crashes • Not suitable for everyone • Not suitable for long distances	[215]
	Public transport	• Reduces pollution and greenhouse gas emissions • No maintenance required by the user • Good alternative for people who are unable to drive or do not have a car • More cost-effective than individual transportation in population-dense areas • Safer than individual transport	[216–218]	• Limited transportation systems in some countries • Can be unreliable • Not convenient for the transportation of bulky or heavy items • Not suitable for remote locations • Safety concerns for vulnerable populations • Health concerns regarding communicable diseases	[216–218]
	Electric vehicles	• Zero tailpipe emissions • Mitigate local air pollution issues • Greenhouse gas emissions over their lifetime are typically lower than those from average gasoline-powered vehicles • Convenient for short distances • Less operational cost than combustion-engine cars • Less maintenance than combustion-engine cars • Can be charged at home • Government subsidies	[156, 219, 220]	• Large initial expense • Battery lifetime issues • Expense of replacement battery • Production of batteries for electric vehicles is more energy intensive than that of combustion engine vehicles • Limited availability • Long charging times	[156, 219, 220]
	Fuel cell technologies	• High theoretical energy efficiency (> 80%) • Environmentally friendly • Lightweight • Compact • Quiet	[221–223]	• Emerging, prototype stage • Expensive to manufacture due to expensive catalysts • Challenging to transport hydrogen gas • Environmental impact of current hydrogen production from fossil sources	[221–223]
Information and Communication Technology	Quantum computing	• Drastically increase computing efficiency • Require significantly less energy than conventional computing • Work with high levels of encryption	[159, 224, 225]	• Require a new algorithm for every type of computation • Typically require extremely low temperatures (15 mK), which are energy-intensive and difficult to maintain • Very expensive • May be able to decrypt all existing codes on the internet, which can cause internet security problems	[224, 225]
	Ambient condition superconductivity	• Potential to eliminate most heat losses because of resistance-free electrical conduction	[161]	• Do not exist yet	[161]
Chemicals	Electric arc furnaces	• Ability to produce all ranges of steel grades • Exceptional metallurgical control • Low pollutions • Powered by electricity, which can be renewable	[226, 227]	• Expensive • High electricity demand • Poor thermal efficiency • Slag production	[226, 227]
	Electrolysis processes	• Pure products • Continuous process, high efficiency • Can be used on a large scale • Powered by electricity, which can be renewable	[228, 229]	• Can be energy intensive (chloralkali process, purification of metals) • Products may release large amounts of liquid waste	[228, 229]
	Electrocatalysis	• Efficient • Cost-effective • Low capital expense • Can use electricity powered by renewable sources • Promising method for storing renewable electricity	[230, 231]	• Catalysts can be very expensive • Many processes are emerging and not viable yet	[230, 231]
	Direct air capture of CO_2_	• Net-carbon-negative • Can be operated in many different locations as long as there is a geologic formation where the captured carbon dioxide can be stored • Low land use	[171]	• Very expensive • Potential for hazardous leaks of concentrated CO_2_ • Energy-intensive • More development needed	[171]
	Artificial photosynthesis	• Environmentally friendly • Uses solar energy • Converts sunlight into renewable, clean, and sustainable energy feedstocks	[175, 232–239]	• More development needed • Expensive noble-metal catalysts • Expensive, high-purity semiconductor light absorbers • Durability issues	

**Long-term solutions.** A sustainable future requires a complete shift to net-zero and net-negative carbon technologies to meet global human needs. The sun is the most abundant, globally scalable, affordable, distributed, and cleanest source of energy, but the local intermittency of solar radiation causes power generation fluctuations that require storage of solar energy on a large scale. Long-term successor technologies address sunlight capture, conversion, and storage of solar energy. While battery technology has made great strides and modern life is inconceivable without batteries [240, 241], global-scale storage of energy requires more cost-effective solutions [242]. Only solar fuels, i.e. storage of sunlight in chemical bonds, have the potential to become a widespread primary energy source [127, 172–175, 242, 243]. Most mature to date are solar-to-hydrogen technologies that consist of water electrolyzers powered by excess grid electricity, for example from wind parks [244]. Hydrogen evolution systems that are powered by solar photovoltaics [245] and hydrogen storage integrated within hybrid renewable energy systems are emerging [246]. Research continues on photovoltaic materials to enhance sunlight conversion efficiency [247]. Third generation solar cells, i.e. multi-junction, dye sensitized, quantum dot, perovskite, and organic solar cells, have the potential to achieve 31–41% power efficiencies [248]. Space-saving bifacial [249] and floating [250] designs are in development [251] to avert a land crisis.

New catalytic materials and processes must urgently be developed to store solar energy after sunlight capture and conversion to electricity. Photo- and electrocatalysts must possess high activity and selectivity for fuel generation from abundant feedstocks, such as water, carbon dioxide, and nitrogen. Only catalysts and processes that can be scaled up to the massive global scale outlined above have potential for viability. Solutions that are amenable to decentralized use offer additional benefits by saving transportation costs and associated emissions.

Solar energy is by far the largest exploitable renewable energy resource, delivering 4.3 × 10^20^ J per hour to Earth’s surface [252]. That means that more energy from sunlight strikes Earth in 90 min than the total world primary energy consumption (5.8 × 10^20^ J) was in the entire year 2019 [2, 252]. In other words, the sun supplies greater than 6,000 times more energy to Earth than the total current world primary energy consumption. Ergo, sustainable successor technologies must utilize solar energy if they are to be able to meet the enormous global scale. Because of the abundance of solar energy, future sunlight capture, conversion and storage technologies have the potential to supply significantly more energy than what is currently produced and do so without greenhouse gas emissions. Most global human needs are connected to energy supply (see above), which creates tremendous opportunities for innovations in the fuels and chemical industries for improved sustainability, predicated on fundamental science and engineering. Making affordable, clean energy available in all corners of the world, i.e. solving the global energy problem, will provide resource security and sustainability for everyone, and enhance environmental and social justice.

## Catalysis Solutions

**Catalyst design and development.** Catalysts encompass biocatalysts, homogeneous catalysts, bulk catalysts and nanocatalysts. Solid catalysts are more robust albeit much less understood than molecular or enzymatic catalysts [126]. Nanomaterials are often better catalysts than bulk solids because of maximized surface area and quantum electronic effects [100]. Electrocatalysts interconvert electrical and chemical energy, whereas thermochemical catalysts transform heat and internal energy into chemical energy. New synthetic and computational methods are needed for the development of advanced catalysts that are robust, effective, selective, and globally scalable [100–102, 104, 123, 126, 230, 253–257]. Detailed mechanistic understanding during turnover, i.e. through *operando* experiments, together with catalyst property–performance relationships, including use of best practices, benchmarking performance assessments, and atomistic understanding of catalyst surfaces, are the foundation of accelerated catalyst discovery through rational materials design [167, 253, 258–271]. Developing advanced catalysts can be challenging because many often-interconnected factors affect catalytic activity and selectivity, such as catalyst surface structure, morphology, composition, electronic structure, chemical environments, and reactor or electrolyzer design. Because of this complexity, density functional theory calculations and machine learning can aid the understanding of how surface species bind to catalysts [272–274]. Atomistic electronic structures control the binding of reaction intermediates at catalyst surfaces, whereas ensemble effects govern the geometric arrangement of surface atoms [122]. Heterogeneous catalysts may additionally benefit from catalyst–support interactions, complicating materials further [275]. In general, catalytic activity can be enhanced by increasing the number of active sites or by increasing the intrinsic activity of each active site [230]. Both strategies can simultaneously be employed. Nanostructuring, using high-surface area supports [276], tailoring morphologies, and using higher catalyst loadings typically increase the number of active sites [230]. A tradeoff exists for catalyst loading between maximizing the number of accessible active sites and minimizing mass and charge transport limitations, optical obscuration in photoelectrocatalytic systems [277], and catalyst cost [278–280]. For noble metal electrocatalysts in market-scale stack electrolyzers, a major cost driver is the high price of electrocatalysts, which are applied at high loadings of > 3 mg cm^–2^ [281].

**Overall process development.** Catalyst development is critical for the optimization of catalytic technologies. In addition, the catalysis and reaction engineering of process design parameters, particularly mass [282] and heat transport [283–289], must be optimized for highest sustainability and economic viability, which dictates that markets are large and profitable. Each catalytic system has its own characteristic engineering controls that enhance the overall efficiency of the system at the scale of investigation; upscaling to industrially relevant levels poses additional challenges [290–292]. In electrocatalytic systems, electrolyte engineering plays a key role for performance [293, 294]. Electrochemical processes face two distinct barriers: cost and catalyst development. If a steady supply of renewable electricity is available at 0.04 U.S. dollars per kWh, catalytic electrosynthesis of chemicals is most likely to effect the largest reduction in carbon emissions compared to other methods, such as thermocatalytic chemical manufacturing [295]. The electricity cost of 0.04 U.S. dollars per kWh was estimated based on systems that reach an overall energy conversion efficiency of at least 60% [295], which implies that the development of highly active, selective, and stable catalysts is critical for realizing economic feasibility of electrocatalysis technologies. Once a suitable, nonprecious, and nontoxic catalyst material exists, catalyst cost may become second to electrolyzer and operational expenses [296]. Likewise, catalyst cost is not the driver in biocatalytic processes [297]. In thermocatalytic processes, water removal rates enhanced conversion levels and led to reactor designs that accelerated water removal [298, 299]. Likewise, reactor design has been utilized to steer electrocatalytic product formation by enhancing reactant and electrolyte mass transport [300–304]. Photocatalysis has its own reactor design strategies, which often entail the integration of light absorbers and electrocatalysts into photoelectrocatalytic devices; other configurations are photocatalytic membrane reactors or traditional photocatalytic arrangements [305]. Important process parameters for photocatalytic systems are the locus of catalyst materials (immobilized vs. suspended), light source (wavelength, intensity), and reactor style (batch vs. hydrodynamic) [305–308]. In biomass systems, optimization of gas–solid heat transfer rates and minimization of particle agglomeration improved overall conversion [309, 310]. Process intensification is essential for enhanced performance and concomitant sustainability, even at the laboratory level, but upscaling to industrial scale requires a more complex systems-level optimization approach, as described in Sect. 3 (Fig. 4).

**Water splitting.** Water splitting is the light- or electricity-driven production of oxygen and hydrogen from water [126]. Hydrogen is a fuel with a particularly high gravimetric energy density, rendering it a transportable energy carrier. Hydrogen is indispensable in the Haber − Bosch process to make ammonia for agricultural fertilizer, in the chemical industry to produce methanol, in hydrodealkylation, hydrocracking, and hydro-desulfurization, in the food and cosmetics industries to hydrogenate fats and oils, and as flushing gas in the semiconductor industry [100]. Key challenges to sustainability are cost and CO_2_ emissions associated with H_2_ production by conventional fossil fuel steam reforming, which releases 9 − 12 tons of CO_2_ per ton of H_2_ produced, depending on the fossil feedstock [311]. Therefore, more sustainable alternative processes are needed for green hydrogen generation.

*Electrocatalytic water splitting.* Electrocatalytic water splitting consists of water oxidation at the anode, which liberates protons and electrons, and hydrogen production from these protons and electrons at the cathode [126]. Electrocatalytic water oxidation can proceed through a four-electron, four-proton pathway to dioxygen, or a two-electron, two-proton process to hydrogen peroxide at the anode [100, 104, 126]. Hydrogen peroxide formation is kinetically easier, but H_2_O_2_ is thermodynamically less stable than oxygen, creating a selectivity challenge for the less oxidized product [100, 104, 312, 313]. Synthesis of hydrogen peroxide for water purification via electrocatalysis is advantageous compared to conventional H_2_O_2_ synthesis because of lower energy demand and obviation of large industrial facilities [312, 314]. Methods for decentralized H_2_O_2_ electrosynthesis create opportunities for providing purified water to remote regions of the world [315]. Metal-containing and metal-free catalysts have been developed to selectively oxidize water to hydrogen peroxide at faradaic efficiencies ≥ 90%; however, the activity remains low across all catalysts [316–319]. Electrocatalytic oxygen evolution from water is the bottleneck in hydrogen evolution from water [126]. Earth-abundant nanocatalysts that operate in alkali electrolyte oxidize water to oxygen with 100% efficiency and low overpotentials [126, 320–323]. Nevertheless, electrolyzers powered by wind electricity operate at high current density conditions and ergo require acidic electrolytes for mass transport reasons [104]. In acid, the most efficient and stable oxygen evolution electrocatalysts contain iridium and ruthenium [104], creating an economic barrier for upscaling because of the cost and availability of noble metals [124, 324]. Commercial water electrolyzers to store excess wind electricity in chemical bonds do exist [244], but their catalysts consist of noble metals (platinum, iridium, and ruthenium), restricting their profitability on a global scale. As a result, green hydrogen currently has a miniscule market share of 0.1% of global dedicated hydrogen production [164]. Water splitting in neutral or near-neutral pH conditions simplifies the system by mitigating corrosion issues that may exist in strongly acidic or alkaline conditions, but coevolution of explosive mixtures of hydrogen and oxygen remains a challenge [104]. The best performing catalysts in neutral electrolytes require noble metals such as platinum, iridium, and ruthenium [325–327]; cobalt oxide also performs well [328]. Another significant drawback of neutral or near-neutral pH conditions is the low concentration of protons and hydroxide ions, which leads to detrimental charge transfer losses and thus limits performance [104]. Seawater oxidation is the next frontier in water oxidation [104]. The main challenges are competing electrochemical halogenide oxidations and catalyst inactivation by complex formation with the ions dissolved in seawater (Na^+^, Cl^–^, Br^–^, Ca^2+^, Mg^2+^, CO_3_^2–^) [104]. A few seawater oxidation catalysts have been reported [329–331], but none appear viable to date on a large scale. Technoeconomic analysis established that an integrated water splitting device must have a combined overpotential for oxygen and hydrogen evolution electrocatalysis of less than 0.45 V at a current density of 10 mA cm^–2^ [332].

*Solar hydrogen production.* The sun is by far the largest exploitable renewable energy resource (see above). Therefore, solar hydrogen production is an attractive option. Because of the large interest in this field, best practices and benchmarking performance protocols are available [269, 333]. Solar water splitting encompasses three device designs: photocatalytic, photoelectrocatalytic, and photovoltaic–electrocatalytic hybrid systems [176]. The simplest engineering design is photocatalysis, which harnesses photocatalysts dispersed in water for hydrogen production under sunlight irradiation [334–336]. However, even with the generous assumption of 100% quantum yield for incident-photon-to-carrier conversion, most catalysts possess a theoretical solar-to-hydrogen conversion efficiency of 18% [334]. Thus, and because of inevitable losses in real materials, typical solar-to-hydrogen conversion efficiencies are below 2% in photocatalytic water splitting systems [334]. In contrast, photoelectrocatalysis separates hydrogen and oxygen evolution catalysis, and photocathode and photoanode light absorber materials, using an ion-conducting membrane. This separation of the two half reactions resulted in significant improvements in solar-to-hydrogen conversion efficiencies, with some devices achieving up to 13% [337]. Photoanode materials consisted of toxic and expensive semiconducting gallium arsenide [338–340], impeding worldwide scaleup. Most efficient to date are photovoltaic–electrochemical hybrid systems, which are essentially solar driven electrocatalytic devices [333]; solar-to-hydrogen efficiencies of > 30% have been observed [341]. Even with such high efficiency, current solar hydrogen production systems are not economically viable. System-level analyses suggest the following technoeconomic targets for solar-to-hydrogen systems: an active component lifetime of 7 years and an efficiency of ca. 25% [342].

**Ammonia production.** For more than one hundred years, the Haber–Bosch process has been providing ammonia to the world; ammonia is an essential chemical for fertilizer production and many other industries [343]. This highly efficient thermocatalytic process requires high temperatures (300–500˚C) and pressures (150–200 atm) to convert a gas stream of hydrogen and nitrogen to ammonia. Despite decades of optimization, the Haber–Bosch process consumes 3–5% of the world’s natural gas for H_2_ production, takes 1–2% of the world’s total energy consumption, and releases an amount of CO_2_ that accounts for 1.5% of all greenhouse gas emissions [344]. Ammonia demand continues to increase for agricultural fertilizer production to provide a stable and affordable food supply for a still growing world population and as precursor for urea for automotive combustion engine emission reduction [345]. Sustainable ammonia production is therefore coveted. The U.S. National Academy of Engineering identified management of the nitrogen cycle as one of the 14 Grand Challenges for Engineering in the 21st Century [346]. The scientific understanding of sustainable ammonia synthesis must be improved to form the foundation for alternative processes, although it is unlikely that a thermochemical process would be more energy efficient than the Haber-Bosch process; reduction of the carbon footprint of thermochemical ammonia production will be driven by using sustainable energy and reactants, i.e. solar hydrogen [344]. Ammonia can serve as chemical storage for renewable hydrogen [347].

*Electrocatalytic NH*_*3*_*production.* Ammonia electrosynthesis can be less energy-intensive than the Haber–Bosch process [140, 348]. The cost of electricity, conversion rate, and conversion efficiency dominate the cost of produced NH_3_ [348], suggesting that electrocatalyst materials are the main cost driver. Electrocatalytic reduction of nitrogen and nitrates obviates the high temperatures and pressures and large industrial facilities that the Haber–Bosch process requires [140]. Major challenges in electrocatalytic ammonia production are the high stability of the nitrogen triple bond and competing hydrogen evolution [349]. Although computational modeling advanced the understanding of nitrogen fixation and ammonia electrosynthesis [350–358], experimental efforts resulted in solid-state catalysts with limited performance for aqueous nitrogen reduction [356, 359]. Faradaic efficiencies for ammonia of ca. 80% have been observed, but at the expense of conversion yields [360–363]. Materials that achieved yields of ≥ 100 µg mg_cat_^–1^ h^–1^ lacked selectivity, with faradaic efficiencies for NH_3_ below 40% [362–364]. Nitrate reduction is another strategy for sustainable ammonia production [138]. The bond dissociation energy of the nitrate N–O bond is significantly lower than that of the N≡N bond; thus, ammonia formation is easier from nitrate [365, 366]. In addition, competing hydrogen evolution is less of a challenge in nitrate reduction [140]. Nonaqueous lithium-mediated ammonia production does not suffer from competing hydrogen evolution [367, 368]. Electrochemical nitrate to ammonia conversion requires the transfer of 9 protons and 8 electrons, leading to undesired byproducts such as NO_2_^–^, N_2_, and N_2_H_4_, which require the transfer of less charges [140]. Catalyst design and electrolyte engineering are at this time the premier research directions [140, 369]. Efficiencies of ≥ 90% and ammonia yields up to 100–10,000 µg h^–1^ have been achieved for nitrate reduction electrocatalysis [365, 370, 371]. Technoeconomic analysis suggests that nitrate to ammonium nitrate conversion will be most viable; a total cell potential of ca. 2.2 V and cell efficiency of ≥ 60% are targets if renewable electricity cost falls below 0.02 U.S. dollars per kWh [369].

*Photocatalytic NH*_*3*_*production.* Photocatalytic approaches to ammonia synthesis are fraught with the same issues as electrocatalytic systems, namely the high stability of the N≡N bond and competing hydrogen evolution. Select photocatalysts have shown ammonia yields greater than 180 µmol g^–1^ h^–1^ under visible light radiation for dinitrogen fixation [372–375]. Photocatalytic nitrate reduction reached 97% selectivity but only ≤ 100 µmol NH_3_ was generated over the course of 24 h [376].

**Carbon dioxide reduction and conversion.** Turning climate-damaging CO_2_ into fuels and upgraded chemicals is a holy grail of catalysis science. Electrocatalytic carbon dioxide reduction and thermocatalytic CO_2_ hydrogenation are major areas of research and development.

*Electrocatalytic CO*_*2*_*reduction.* Sustainable technologies for CO_2_ conversion into single upgraded products will be essential to mitigate anthropogenic climate change [101]. The electrocatalytic reduction of CO_2_ to fuels is a promising successor technology that seeks to close the linear fuel consumption and emission process by looping CO_2_ into liquid fuel generation [101]. At ambient temperatures and pressures, using only CO_2_ and water as feedstocks, CO_2_ reduction aims to produce high-value commodity chemicals, which currently rely on fossil-fuel-powered energy-intensive processes [101]. The central challenge with CO_2_ reduction is the development of catalysts that will effectively produce a single product over extended periods of time [101]. Desired C_1_ or C–C coupled products require 6–18 or more electron and proton transfers, whereas the less desired products (H_2_, CO, and formate) require the transfer of only two electrons and protons. Likewise, standard potentials of many CO_2_ reduction reactions are within approximately 200 mV of one another, and proton reduction to hydrogen is thermodynamically easier, making selectivity towards one desired product inherently difficult [101]. Typically, the highest reported faradaic efficiencies are achieved for CO and formate production [101]. Gold clusters and gold nanomaterials electroreduced CO_2_ to CO with faradaic efficiencies up to 100% [377, 378]. High faradaic efficiencies and activities for formic acid production were observed with tin or bismuth catalysts [277, 379]. Copper is the only single metal catalyst that can produce C–C coupled products at high activities due to its moderate adsorption energy of CO intermediates at copper surfaces [380]. However, copper cathodes suffer from selectivity issues; mixtures of C_1_ and C_2+_ products are almost always obtained [381]. Separation costs are detrimental to commercial viability at an industrial scale [101]. Multimetallic copper-containing materials electroreduced CO_2_ to ethanol with faradaic efficiencies of 40–60% [382–384]. The formation of C–C coupled products is determined by the first two-electron reduction step to HCOO^–^ or CO. Bound HCOO^–^ prevents further C_2+_ product generation, whereas bound CO can lead to C–C coupling [167]. As a result, direct electrochemical CO reduction can be utilized to produce value-added chemicals with higher selectivity towards liquid oxygenated C_2+_ products and little to no H_2_ evolution, together with efficient technologies that generate CO from CO_2_ [382, 385]. Implementation of CO_2_ and CO reduction catalysts into gas diffusion electrodes increased the overall selectivity and energy efficiency [386, 387]. The technoeconomic threshold for industrial feasibility is at 300 mA cm^–2^ and 0.5 V overpotential with 70% faradaic efficiency for a single C_2+_ oxygenate product [388, 389].

*Photocatalytic CO*_*2*_*reduction.* Photocatalysts have limited stability, with many being stable for only a few hours [390]. Photocatalysts capable of converting CO_2_ into methane and methanol have been reported [391]. Photocatalytic materials that showed C_2+_ selectivity up to 100%, but with low yields, have been published [392, 393]. Experts have established that photocatalytic CO_2_ reduction systems must reach a current density above 80 mA cm^–2^ or CO_2_ conversion efficiency of 22% to be economically viable [394].

*Thermocatalytic CO*_*2*_*upgrading.* Thermocatalytic hydrogenation of CO_2_ to valorized chemicals and fuels occurs mainly via CO_2_-modified Fischer-Tropsch synthesis or methanol-mediated processes [395–399]. Alternatively, the reverse water gas shift reaction is employed to produce CO, an important feedstock for reductions [400]. Obstacles to sustainability are the purification of captured CO_2_, sourcing of H_2_ (fossil-derived or renewable), catalytic efficiency and selectivity, separation costs of formed products, and energy inputs for hydrogenation reactions [401].

**Carbon capture and storage.** Carbon capture and storage technologies are necessary to remove anthropogenic CO_2_ from the atmosphere and lower future emissions. Carbon capture and storage technologies can decarbonize current fossil fuel supply chains, to offer an immediate “zero-carbon” technology during the global scaleup of renewable energy technologies [402]. Historically, carbon capture and storage approaches focused on biological pathways, such as large-scale afforestation [403, 404] and bioenergy with carbon capture and storage [405, 406]. The major concern with biological methods is whether they are adequate for removing CO_2_ from the oceans and atmosphere at a rate of 10 gigatons per year by the year 2050, to limit climate change to around 1.5 degrees Celsius, a goal set by the Paris Accords [5]. Further, CO_2_ storage in forests is, in general, temporary because stored CO_2_ is released when trees die. At the enormous global scale outlined above, afforestation and bioenergy with carbon capture and storage compete with land and water needs [407].

Direct air carbon capture and sequestration technologies are a more sustainable approach. Using sorbent materials, CO_2_ molecules are captured by physisorption, chemisorption, or dissolution from air, through adhesive forces between CO_2_ and sorbent surfaces [171]. After CO_2_ capture, sorption materials are treated (typically by heat) to release the captured CO_2_ into geological storage sites for further utilization [171]. Integration of thermochemical water splitting with CO_2_ direct air capture has been demonstrated [408]. Recently, three major direct air carbon capture and sequestration companies have emerged–Carbon Engineering, ClimeWorks, and Global Thermostat–which claim to be capable of capturing ≥ 1 megaton CO_2_ per year, which is at a relevant scale for global decarbonization and equivalent to the CO_2_ capture and sequestration capability of 40,000 photosynthetic plants [409]. Carbon Engineering estimates their overall operating plant costs to be in the range of 94–232 U.S. dollars per ton CO_2_ [410]. ClimeWorks has two commercial facilities in Hinwil, Switzerland, and in Hellisheiðarvirkjun, Iceland, which are capable of capturing 50 and 900 tons CO_2_ per year, respectively [411]. In 2018, ClimeWorks reported their operating cost as 600 U.S. dollars per ton CO_2_, with the prediction that expanding operations and innovations would drop costs to 100 U.S. dollars per ton CO_2_ within 5–10 years. Experts predict that sequestering CO_2_ at a cost of 100 U.S. dollars per ton CO_2_ to offset vehicle carbon emissions would add about 0.22 U.S. dollars per liter of fuel [411]. As with many technologies, the economics of CO_2_ capture and sequestration depend on factors that vary by location, including the cost of energy and access to government subsidies. The opportunity to convert emissions into fuels, provided that viable catalysts and processes exist, creates an economic incentive, in addition to the established environmental motivation, for carbon capture technology development and commercialization.

**Hydrocarbon oxidations.** Selective oxidations of hydrocarbons to desired oxygenates are very important industrial processes [412] that must become more sustainable.

*Electrocatalytic hydrocarbon oxidations.* Methane can serve as a chemical feedstock with a smaller carbon footprint than current fossil feedstocks [413–415]. Methane can be used for bottom-up manufacturing of plastics, industrially important organic chemicals, and fuels; main challenges are overoxidation of methane to CO_2_ and difficult C–H activation [413–415]. Electrocatalytic methane oxidation with renewable electricity has attracted much interest [416–418]. Metal oxides, hydroxides, and nanoclusters, as well as single metal catalysts have been investigated for methane oxidation in neutral or alkaline conditions [419–422]. Metal oxides performed best and achieved overall cell efficiencies of ≤ 60%, selectivities of ≤ 90%, and formation rates of ≤ 2 µmol h^–1^ cm^–2^ at a current density of 4.0 mA cm^–2^ [423]. However, to compete with conventional steam-reforming technology for methane to methanol conversion, economic viability targets are at least 70% conversion efficiency, 200 cm^2^ cell area, 100 mA cm^− 2^ current density, and 1.0 V cell voltage [423, 424]. For future commercialization, new catalysts are needed; therefore, fundamental studies aimed at understanding electrooxidation mechanisms and catalysts will provide the necessary framework for accelerated catalyst and process development.

*Photocatalytic hydrocarbon oxidations.* Advances have been made in photocatalytic systems despite the challenges of C–H activation in molecules with high C–H bond dissociation energies and overoxidation issues [425]. The highest performing materials for the partial oxidation of methane to upgraded products achieved a selectivity of over 50%, albeit with low overall conversion efficiencies and production rates, inhibiting near-term commercialization [426–428].

*Thermochemical hydrocarbon oxidations.* Heat-driven hydrocarbon oxidations play an important role in many technological processes, such as combustion in engines and furnaces, flame synthesis of materials, aerobic partial oxidations in industrial petrochemical processes, olefin epoxidations, catalytic combustion, and exhaust gas treatment [429–432]. Although the chemical industry has optimized most processes, the main barriers against sustainability are high-temperature and high-pressure requirements and use of fossil feedstocks.

**Methane Pyrolysis.** Methane pyrolysis has emerged as a potential zero-emission hydrogen production method that can bridge the gap between fossil fuel and renewable technologies [433]. The process thermally decomposes methane into hydrogen and solid carbon [433]. Methane pyrolysis requires only 37.7 kJ per mole H_2_ and is thus thermodynamically more favorable than steam-methane reforming coupled with the water − gas shift reaction, which needs 41.4 kJ per mole H_2_ [433]. Thermal and catalytic methane pyrolysis technologies have emerged, with similar technical challenges that limit industrial application [433–440]. Challenges are high process temperatures, purity of produced hydrogen gas, and separating the solid carbon from the gas phase. Use of natural gas instead of pure methane as feedstock is necessary for global scalability; a deep mechanistic understanding of the effects of minor natural gas components on catalyst activity and stability must be developed [433]. In addition to the technical challenges, the market scale for solid carbon poses an economic quandary: 100 megatons of solid carbon would be produced each year, which is five times the amount of what is globally needed today, if methane pyrolysis were to replace current-scale steam-methane reforming [441]. Technoeconomic analysis revealed that methane pyrolysis costs 1.62 U.S. dollars per kg H_2_, which is more expensive than steam reforming (1.10 U.S. dollars per kg H_2_); without a government-stipulated carbon tax, state-of-the-art methane pyrolysis is not economically feasible [442].

**Biomass conversion.** Biofuels can potentially substitute fossil fuels if carbon tax and emissions trading incentives make fossil energy more expensive [443]. Agricultural production and biorefinery plants generate first-generation biofuels, which are produced from sugar, starch, vegetable oil, or animal fats. For first-generation biofuels, food crops are devoted to the production of energy [444]. The U.S. is currently the world’s largest producer of bioethanol with a production of 19.8 billion liters per year, from corn as primary feedstock [445]. Sugarcane is used as the primary feedstock in Brazil, the world’s second-largest producer of bioethanol (17.8 billion liters per year) [445]. However, crop-derived biofuels compete with food availability and animal-feed industries, and thus contribute to food shortages [446]. Second-generation biofuels partially overcome the limitations of first-generation biofuels. The feedstocks of second-generation biofuels are non-food crops, such as residues from agriculture, forestry, and industry, as well as dedicated lignocellulosic crops, short-rotation forestry, and perennial grasses, e.g. switch grass. Second-generation biofuels additionally have advantages regarding land-use efficiency and environmental performance, compared to first-generation biofuels [447]. Most processes and technologies for second-generation biofuels are still at a precommercial stage but could enter the market within 10–15 years if associated investments are made. Cost drivers are growth of non-food crops and processing of biomass waste materials. However, upgrading biowaste feedstocks potentially enables the coproduction of valuable biofuels, chemical compounds, as well as electricity and heat, leading to better energy, environmental and economic performance through engineered biorefinery concepts [448]. Biorefining is the sustainable processing of biomass into a large range of commodity chemicals and energy and may lead to a sustainable biobased society. The most important energy products of biorefineries are gaseous biofuels (e.g., biogas, syngas, hydrogen, biomethane), solid biofuels (e.g., pellets, lignin, charcoal, biochar), and liquid biofuels for transportation (e.g., bioethanol, biodiesel, bio-oil) [449]. Challenges to economic viability are conversion costs, costs associated with water, fertilizer, and land use, and costs associated with harvesting and transporting biomass to conversion plants [450, 451].

*Catalytic approaches.* Efficient catalytic conversion of biomass into chemicals or fuels is a key challenge. In general, biomass is first broken down into smaller molecules using “brute force” high-temperature, high-pressure reaction conditions, after which these small molecule precursors are turned into useful chemicals by catalytic reactions. Biomass contains a mixture of many different functional groups (e.g. alcohols, esters, ethers, and carboxylic acids), which presents a major obstacle for the selective and profitable production of single chemicals [452]. Additionally, the high oxygen content of biomass poses challenges for the conversion of biomass into hydrocarbon fuels with high energy density; reducing the oxygen content and C–C coupling between parent monomers to obtain the appropriate molecular weight and volatility of the final hydrocarbon product are required for use of biofuels in the transportation sector [453].

Most state-of-the-art biomass conversion processes are fraught with issues, such as low product yields, harsh reaction conditions, high processing cost [454], difficult separations, and poor compatibility with existing infrastructure [455]. Another disadvantage of biomass conversion is that an external hydrogen source is necessary [453], whose sustainability impact can be improved by usage of green hydrogen [456–458], or minimizing the amount of external hydrogen, which has been demonstrated for the integrated catalytic conversion of γ-valerolactone [459]. Furfural is one of the most studied biomass compounds. It is typically produced by acid-catalyzed hydrolysis and dehydration of hemicellulose, and it can be used to obtain furfuryl alcohol and/or 2-methyl furan, which are an adhesive intermediate and a promising biofuel candidate, respectively [460], via thermochemical catalysis [461].

Some of the most well-developed strategies for producing bioderived jet fuel are lignocellulose gasification [455], Fisher-Tropsch synthesis [462–466], and triglyceride hydrodeoxygenation [467–471]; technologies that efficiently leverage lignocellulose appear to date most promising for the catalytic conversion of biomass into jet fuels [455]. Some of the commercially used or near-commercial technologies for second-generation biomass conversion include: biomass-derived jet and diesel fuel from vegetable oils and ethanol, small scale production of renewable jet and diesel fuels from landfill gases via Fischer-Tropsch synthesis, catalytic conversion of carbohydrates into gasoline and aromatics, hydropyrolysis of biomass into gasoline and diesel fuels, and catalytic conversion of food into aromatics [472]. These technologies must be able to compete economically on the global scale with well-established petroleum technologies, which remains a challenge [472].

Catalyst design for biomass conversion must leverage a deep understanding of the complexity of biomass, which consists of mixtures of matter with a multitude of functional groups. Solid catalysts can possess beneficial features [473], such as cooperative catalytic sites (e.g., early transition metals and strong Brønsted acid sites) that promote the cleavage of C–O and C–C bonds, catalyst supports that weaken phenyl adsorption to impede hydrogenation, and optimized active sites that promote hydrogen dissociation (e.g., bimetallic nanoparticles) [474]. The biggest challenges in biomass conversion catalyst development are selectivity for one desired product at high activity and preventing catalyst poisoning [472].

*Biorefineries.* Catalysis will play a crucial role for establishing robust biorefineries that produce upgraded chemicals [475]. Note that biorefineries that produce charcoal or biochar do not require catalysts; they work by a coking process, i.e. heating biomass in oxygen-lean conditions [476]. Catalysts are central in the design of biorefineries, e.g., for gasification to produce energy carriers, such as N_2_, H_2_, or CO for efficient heat and power cogeneration [477], catalytic combustion of woody biomass to produce chemical compounds [478], and liquefaction to high energy bio-oil [479], or direct catalytic conversion of biomass into biofuels [480]. Biorefineries for biomass gasification operate under atmospheric or pressurized conditions. Gasification refers to partially oxidizing and thermally transforming biomass (below 1300 °C) into simple gas-phase compounds (e.g., N_2_, H_2_, or CO). Fluidized bed gasifiers are the most often utilized reactor design, which often operate with oxygen, air, steam, and nickel catalysts [481]. Woody biomass conversion to oily chemicals using heterogeneous catalysis (e.g., CaO, Ni-W_2_C, or acid catalysts) can be performed inexpensively [478]. Use of woody biomass, such as hardwood, coniferous and eucalyptus, as feedstocks have been reported [478]. Liquefied products are often obtained from a combination of catalysis and high-temperature processing for efficient biomass decomposition [482].

*Algal biomass.* Algae are an attractive source for many types of biofuels, including biogas from anaerobic degradation of biomass, bio-diesel from lipids in algal cells, and alcohols or hydrogen from photobiological transformations of algal biomass [483]. Algae produce more biomass than terrestrial fuel crops [484]. With sufficient sunlight, algae consume CO_2_ and organic nutrients in water to photosynthesize organic matter at rates that double algal mass several times a day [484]. Cyanobacteria have a wide range of habitat and found worldwide use as an efficient low-cost dairy wastewater remediation technique by converting dissolved nutrients into algal biomass [485–487]; note that natural cyanobacteria can be toxic [488]. Engineered cyanobacteria have been employed as catalysts for the direct conversion of CO_2_ into ethanol, *n*-butanol, and isoprene [489]. Resources for algae growth do not compete with food production; algae farms require non-arable land, non-potable water, waste nutrient streams, waste carbon dioxide, sufficient sunlight, and a supporting infrastructure to access downstream processing operations [490]. Algae assimilate significant quantities of biogenes from wastewaters because they need high quantities of nitrogen and phosphorus for protein synthesis [483]. Since algae feed on carbon dioxide, algal biomass decarbonizes waste streams, without generating algal CO_2_ emissions. However, the large-scale cultivation of microalgae that is needed to meet the worldwide demand for commodity chemicals would require water, nitrogen, and phosphorus on an enormous scale, which would generate a larger carbon footprint than the CO_2_ benefits of algae [491]. Choice of culture technologies determines the cost-effectiveness of algal biogas production. The application of wastewater as a cultivation medium reduces the cost of water and nutrients, simultaneously remediates wastewater by removal of chemical and biological contaminants, and produces biogas from biomass. Cellulosic biomass can serve as a renewable energy source to produce liquid fuels from breakdown of sugars. Microbial biomass-to-ethanol technology is projected to lead to a selling price of pure ethanol of ca. 0.34–0.50 U.S. dollars per gallon, which is currently on par with conventionally produced ethanol [492]. Algal biomass conversion test sites exist in the U.S. [493] and Europe [494]. More research is needed to validate algae growth rates and biomass compositional analysis upon scaleup, improve cultivation performance, reduce cost for cultivation, reduce cost and increase efficiency of dewatering steps, and identify opportunities for lower-cost carbon, nitrogen, and phosphorus sources [495].

**Waste valorization.** Technologies for waste valorization include waste-to-energy technologies via incineration or fermentation into biogas or bioethanol, and plastics deconstruction, such as upcycling polyolefin waste.

*Waste incineration.* Energy recovery is the goal of waste incineration, to provide heat, electricity, and process steam [496]. Most modern incinerators enable power generation. Solid waste combustion technologies consist of movable grate or fluidized bed reactors, for different types of waste. Movable grate incinerators are for inhomogeneous, as-received waste, whereas fluidized bed incinerators are for homogeneous, pretreated waste [496]. Around the world, movable grate combustion is most widely used because no waste pretreatment and fly ash removal are necessary [496]. In 2019, around 25 million tons of waste were incinerated in 156 thermal waste incineration plants in Germany, the country that utilizes the most waste incinerators worldwide [497]. Advantages of waste incineration are decreased need for landfills and concomitant lower methane emissions from decaying waste in landfills [498]. Drawbacks are high capital and operating expenses [498].

*Fermentation of biomass.* Biogas (mainly methane) production from organic wastes, higher plants, or algal biomass via anaerobic digestion is an attractive route to upcycle waste streams and convert biomass into renewable chemical energy [499–504]. Fermentation can also be used to convert food waste to bioenergy [505], typically bioethanol [506]. Food wastes are a major concern to the environment because of their enormous global scale and associated carbon footprint (see above); rotting food emits methane as a result of microbial breakdown [507]. In fermentation processes, food wastes are pretreated with acid, alkali, thermal, or enzymatic processes, to increase cellulose digestibility, followed by enzymatic fermentation. The production of bioenergy via fermentation reduces food waste, solves food disposal issues, and lowers the carbon footprint of food production and disposal [508]. Engineered biocatalysts, i.e. artificial enzymes, will be vital towards economic viability [506].

*Plastics upcycling.* Plastics pollution seriously threatens biodiversity and marine life [509–513]. Despite growing use of plastics, only 30% are recycled, while the remainder winds up in landfills and the environment, including the oceans [511, 513, 514]. Until now, biomass and waste plastics conversions have largely been studied separately. However, they share considerable structural similarities because of their polymeric nature and types of bonds that link their monomer units; therefore, they deserve a unified view [515]. Depending on the plastics feedstock, catalytic cracking, hydrogenolysis, hydrocracking, metathesis, retro-aldol reaction, reductive cleavage, oxidation-hydrogenation, oxidations, hydrolysis, or solvolysis are useful methods to break down plastics for upcycling [515]. New insights from synergies between biomass and waste plastics conversion technologies open a path for future advances with regard to the efficiency of existing processes and unlocking new technologies, e.g. electrocatalysis [515] and photocatalysis [516]. Electrochemical hydrogenation and hydrogenolysis of furfural is a promising method for production of biofuels and fine chemicals without the need for an external hydrogen source, high pressures, or high temperatures [460, 517]. Hydrofuroin and small quantities of ring-opened products were accessible by electrochemical reduction processes [461]. Recent advances on upcycling polyolefin waste are particularly promising [518–523]. More research on process optimization is needed for economic viability [461]. Another growing field is biomass conversion using sonocatalysis and mechanocatalysis, which produce less waste than conventional techniques, can produce multiple products from a single feedstock, and can operate at mild conditions [524]. More research is needed to enable upscaling of laboratory experiments [524].

## Fundamental Limitations of the Earth System

Fundamental limitations regarding growth of consumption exist because Earth is finite. Planetary boundaries that cannot be transgressed must be identified and quantified, to prevent humanity from creating unacceptable changes to the environment [525]. Enormous technological progress, prosperity, and human health advances resulted from the industrial revolution, with disastrous effects on the environment and climate (see Introduction). Anthropogenic actions have the potential to “push the Earth outside of the stable environmental state of the Holocene” and cause “irreversible and abrupt environmental change” [525]. To prevent these, Rockström et al. have proposed a framework based on ‘planetary boundaries’ that define a safe operating space for human actions, as not to threaten human long-term social and economic development [525]. Nine processes, which are closely interconnected, have the potential to create catastrophic environmental changes when their planetary boundaries are crossed: climate change, rate of biodiversity loss (terrestrial and marine), interference with nitrogen and phosphorus cycles, stratospheric ozone depletion, ocean acidification, global freshwater use, change in land use, chemical pollution, and atmospheric aerosol loading. Critical thresholds for rates of climate change, biodiversity loss, and human interference with the nitrogen cycle have already been exceeded, whereas the thresholds for atmospheric aerosol loading and chemical pollution have yet to be determined [525]. We concluded above that providing affordable and clean energy can solve most challenges of human resource security and sustainability; however, fundamental limits exist for growth because Earth’s resources are finite. Human actions caused a transition from the Holocene to the Anthropocene Epoch, i.e. the human imprint on the global environment rivals global geophysical processes on the Earth System; responsible planetary stewardship addressing the nine boundaries above is, therefore, urgently needed on a global scale to mitigate and adapt to this planetary crisis [526–539].

## Summary

We analyzed how global human needs are connected to energy availability and established that most challenges related to resource security and sustainability can be solved by providing distributed, affordable, and clean energy. Sustainable successor technologies must utilize solar energy because the sun is the largest exploitable renewable energy resource and supplies more than 6,000 times more energy to Earth than the total current world primary energy consumption. Solar electricity provides extraordinary opportunities for electrocatalytic processes for the production of solar fuels and upgraded chemicals. We established that a paradigm shift is needed toward more bottom-up manufacturing from our most abundant, small-molecule feedstocks (water, carbon dioxide, and nitrogen) instead of the current top-down approach of cracking oil and coal for the production of fuels and valorized chemicals.

We outlined in detail the massive scale of global human needs by sector, their relation to energy demand, and interconnectivity beyond the widely recognized water–energy–food nexus. The enormous scale of global needs is the foremost challenge in the transition from fossil fuels to a net-zero or net-negative carbon economy. Sustainable successor technologies must be cost-effective to be viable and deployable on a global scale. They additionally must be ethical, i.e. provide solutions without sacrificing the wellbeing of humans and other life forms. Highlighting the technical, economic, and societal pros and cons of short- to medium-term decarbonization solutions permits an assessment of their practicability, economic viability, and likelihood of widespread acceptance on a global scale. We detailed catalysis solutions that enhance sustainability, along with strategies for catalyst and process development, frontiers, challenges, and limitations, and commented on the need for planetary stewardship because resources on Earth are finite. Solar-powered sustainable successor technologies that are affordable, distributed, and clean have the potential to restore global environmental and social justice.

Electrocatalysis is key for the utilization of renewable electricity. Funding must significantly increase to enable fundamental research needed to accelerate the discovery of new catalysts and climate-friendly, economically competitive catalytic processes. The transition from fossil energy sources to sustainable electrocatalytic technologies is urgent and requires ethical science and engineering solutions to solve humanity’s grandest challenge of providing a livable Earth for future generations.

## Electronic Supplementary Material

Below is the link to the electronic supplementary material.

Supplementary Material 1
